# Extracellular Matrix Remodeling as a Mechanobiological Driver of Breast Cancer Aggressiveness: Comparative Oncology, Multi-Omics, and Artificial Intelligence Perspectives

**DOI:** 10.3390/biology15141164

**Published:** 2026-07-16

**Authors:** João Paulo Ruiz Lucio de Lima Parra, Rodrigo Paolo Flores Abuná, Matheus Henrique Hermínio Garcia, Sandra Maria Barbalho, Maria Angelica Miglino

**Affiliations:** 1 Regenerative Medicine Laboratory “Carlos Augusto Camargo de Souza Baptista”, Universidade de Marília (UNIMAR), Marília 17525-902, SP, Brazil; joaoparra@unimar.br (J.P.R.L.d.L.P.); rodri_abuna@hotmail.com (R.P.F.A.); matheushenrigarcia@gmail.com (M.H.H.G.); 2 Graduate Program in Structural and Functional Interactions in Rehabilitation, School of Medicine, Universidade de Marília (UNIMAR), Marília 17525-902, SP, Brazil; smbarbalho@gmail.com

**Keywords:** extracellular matrix, breast cancer, tumor microenvironment, mechanotransduction, metastasis, comparative oncology, proteomics, metabolomics, lipidomics, artificial intelligence, YAP/TAZ

## Abstract

The extracellular matrix (ECM) is increasingly recognized as an active regulator of breast cancer progression rather than a passive structural scaffold. This narrative review examines how ECM remodeling contributes to tumor aggressiveness through changes in matrix composition, collagen architecture, tissue stiffness, mechanotransduction, stromal permissiveness, immune and metabolic programs, invasion, metastasis and therapeutic response. A structured narrative search of literature published from 1981 to June 2026 was used to support this synthesis. Evidence from breast cancer studies indicates that collagens, fibronectin, laminins, proteoglycans, matricellular proteins, ECM-remodeling enzymes, and matrix-crosslinking pathways regulate integrin–FAK/Src, RhoA–ROCK, PI3K–AKT, MAPK, TGF-β/SMAD, Wnt/β-catenin, and YAP/TAZ signaling. Spontaneous canine mammary tumors are discussed as complementary comparative models that may preserve selected tumor–stroma–ECM interactions under naturally occurring disease conditions while requiring cautious interpretation due to species-specific biological and clinical differences. Proteomics, lipidomics, metabolomics, spatial omics, digital pathology, and artificial intelligence may support ECM-informed biomarker discovery and response prediction. However, translational application requires standardized pathology, reproducible assays, harmonized metadata, external validation, model interpretability, and clinically meaningful endpoints. Overall, ECM-informed comparative oncology is best viewed as a framework grounded in rigorous validation for identifying matrix-defined tumor phenotypes and prioritizing future biomarker and therapeutic strategies.

## Simple Summary

Breast tumors grow and spread within a surrounding tissue environment that can either restrain or support disease progression. This review focuses on the extracellular matrix, the network of proteins and other molecules that supports tissues and helps cells communicate. In breast cancer, this network can be remodeled, making the tissue stiffer, reshaping cell behavior, supporting invasion, altering immune responses, and contributing to treatment resistance. We also discuss naturally occurring mammary tumors in dogs as complementary comparative models. These tumors can help researchers study selected tumor–tissue interactions under real disease conditions, but they should not be considered direct copies of human breast cancer. The review also examines how protein analysis, lipid and metabolite profiling, spatial tissue methods, digital pathology, and artificial intelligence may help identify matrix-related features associated with aggressive disease and treatment response. However, these tools require standardized samples, reliable measurements, careful data integration, and independent validation before they can support clinical decisions. Overall, this review presents the extracellular matrix as an active regulator of breast cancer progression and as a validation-dependent source of future measurable signs of disease behavior and treatment-guiding information.

## 1. Introduction

Breast cancer progression is not determined solely by genetic and phenotypic alterations in malignant epithelial cells but also by dynamic interactions with the tumor microenvironment (TME). Within this microenvironment, the extracellular matrix (ECM) has emerged as a central regulator of tumor behavior, providing biochemical, biomechanical, and spatial cues that influence proliferation, invasion, angiogenesis, immune modulation, metabolic adaptation, and therapeutic response [1,2,3,4,5,6,7]. From a broader cancer-biology perspective, these ECM-dependent functions intersect with multiple malignant capabilities and support the current view of the tumor-associated ECM as an active regulatory axis in cancer progression and metastasis [5,7]. Therefore, ECM remodeling should not be interpreted merely as a structural consequence of malignancy. Rather, it represents an active, reciprocal process in which tumor cells, cancer-associated fibroblasts, immune cells, endothelial cells, and matrix-remodeling enzymes reshape tissue architecture and create microenvironmental conditions that favor tumor aggressiveness.

A key consequence of tumor-associated ECM remodeling is the emergence of stromal permissiveness. In breast cancer, degradation of the basement membrane, altered deposition and alignment of fibrillar collagens, fibronectin accumulation, lysyl oxidase (LOX)-mediated crosslinking, matrix metalloproteinase (MMP)-driven proteolysis, and cancer-associated fibroblast activation collectively modify tissue stiffness, ligand availability, growth-factor release, and invasion tracks [3,8,9,10,11]. These changes transform the ECM from a homeostatic scaffold into a mechanobiological signaling platform. Tumor cells sense matrix stiffness and composition mainly through integrins and focal adhesion complexes, activating FAK/Src, RhoA–ROCK, PI3K–AKT, MAPK–ERK, TGF-β/SMAD, Wnt/β-catenin, and Hippo/YAP/TAZ signaling pathways [9,11,12,13]. These pathways converge on phenotypes associated with epithelial–mesenchymal transition (EMT), survival under mechanical and metabolic stress, invasive migration, immune escape, and resistance to therapy. Thus, ECM remodeling provides a mechanistic bridge between tissue architecture and malignant cell behavior.

The molecular composition of the tumor ECM further contributes to this process. Structural ECM proteins, including collagens, fibronectin, laminins, and elastin, regulate tissue mechanics, polarity, adhesion, and basement membrane integrity. Proteoglycans and glycosaminoglycan-associated molecules modulate growth-factor sequestration, inflammation, hydration, and cell migration. Matricellular proteins, such as tenascin-C, periostin, osteopontin, SPARC, and thrombospondins, act as context-dependent signaling regulators that integrate mechanical stress, stromal activation, angiogenesis, immune modulation, and metastatic niche formation [2,4,8,11,14,15,16]. Importantly, these ECM components do not act in isolation. Their biological impact depends on tumor subtype, stromal composition, spatial organization, enzymatic remodeling, and crosstalk with immune and vascular compartments. This interpretation is consistent with the matrisome framework, which distinguishes core structural ECM components from ECM-associated glycoproteins, proteoglycans, ECM-affiliated molecules, and remodeling regulators [14,16]. This complexity supports the notion that ECM signatures may serve as integrated readouts of tumor aggressiveness rather than isolated biomarkers. Because ECM signatures emerge from spatially organized and temporally evolving interactions among malignant cells, stromal cells, immune components, vasculature, and matrix-remodeling enzymes, their interpretation requires models that preserve tumor–stroma–ECM crosstalk under biologically relevant conditions. This need provides a rationale for incorporating comparative oncology into ECM-informed breast cancer research. Spontaneous canine mammary tumors are relevant in this context because they arise naturally, develop in immunocompetent hosts, and may preserve aspects of tumor–matrix co-evolution that are difficult to reproduce in highly reductionist models [17,18,19,20,21,22]. Their use, however, requires cautious interpretation because species-specific differences in molecular subtype distribution, hormone receptor status, reproductive history, treatment exposure, tissue processing, and histopathological classification may influence translational relevance [17,18,19,20,22].

To translate this biological complexity into measurable and predictive information, recent advances in ECM-focused proteomics, matrisome profiling, lipidomics, metabolomics, single-cell analysis, spatial omics, digital pathology, and artificial intelligence (AI) have expanded the ability to characterize tumor–matrix interactions at molecular, cellular, and spatial scales [14,23,24,25,26]. These approaches may help identify ECM-associated signatures of invasion, immune exclusion, metastatic potential, and treatment response. However, their clinical and comparative value depends on rigorous standards for sample preparation, metadata annotation, data interoperability, cross-cohort reproducibility, external validation, model explainability, and bias control [25,27,28,29]. Without such standards, ECM-based multi-omics and AI models may remain technically sophisticated but clinically fragile.

Several reviews have addressed ECM remodeling, mechanotransduction, tumor stroma, canine mammary tumors, or computational oncology as separate topics. However, there remains a need for an integrated narrative synthesis that connects ECM molecular architecture and mechanobiology with spontaneous comparative oncology and emerging predictive platforms. Therefore, this review examines ECM remodeling as a mechanobiological determinant of breast cancer aggressiveness, with emphasis on three central questions: how ECM composition and mechanics generate stromal permissiveness and invasive phenotypes; how spontaneous canine mammary tumors can inform conserved and divergent ECM mechanisms in comparative oncology; and how ECM-centered multi-omics and AI-based approaches may support biomarker discovery, patient stratification, and treatment-response prediction. By integrating these axes, we aim to provide a critical and translational framework for understanding the ECM not only as a structural component of breast tumors but also as an active regulator of malignancy and a source of clinically relevant information for biomarker development and treatment-response assessment.

## 2. Search Strategy and Scope of the Narrative Review

This article was designed as a narrative review, rather than a systematic review or meta-analysis, to provide a critical and integrative synthesis of extracellular matrix (ECM) remodeling in breast cancer aggressiveness, with emphasis on mechanobiology, spontaneous canine mammary tumors as comparative models, ECM-centered multi-omics, artificial intelligence (AI), predictive biomarker platforms, and therapeutic translation.

A structured narrative literature search was performed using PubMed/MEDLINE, Scopus, Web of Science, and Google Scholar, covering studies published from 1981 to June 2026. The broad temporal range was used to include foundational studies in ECM and tumor microenvironment biology, whereas recent publications were prioritized for rapidly evolving areas such as ECM proteomics, spatial omics, digital pathology, AI-based prediction, and biomarker validation. No formal language restriction was applied at the database-search stage; however, the final synthesis prioritized peer-reviewed articles available in English or with sufficient English-language bibliographic and methodological information to allow accurate interpretation and citation. The following search terms and their combinations were used: “extracellular matrix”, “ECM remodeling”, “breast cancer”, “tumor microenvironment”, “mechanotransduction”, “matrix stiffness”, “collagen alignment”, “lysyl oxidase”, “integrin signaling”, “FAK”, “RhoA ROCK”, “YAP TAZ”, “proteoglycans”, “matricellular proteins”, “matrisome”, “canine mammary tumor”, “canine mammary carcinoma”, “comparative oncology”, “spontaneous tumor model”, “proteomics”, “lipidomics”, “metabolomics”, “spatial omics”, “digital pathology”, “artificial intelligence”, “machine learning”, “predictive biomarker”, and “treatment response”. Because this was a narrative review, the search and selection process was not intended to generate a PRISMA-based workflow, a quantitative pooled estimate, an independent duplicate screening process, formal evidence grading, or a risk-of-bias assessment.

Records identified through database searches and citation tracking were screened at the title and abstract levels for relevance to the review’s scope. Potentially relevant articles were then assessed at the full-text level. Studies were included when they contributed mechanistic, comparative, methodological, or translational evidence related to at least one of the following axes: (i) ECM composition, remodeling, stiffness, and mechanotransduction in breast cancer; (ii) stromal permissiveness, invasion, angiogenesis, immune modulation, metabolism, and therapeutic resistance; (iii) conserved and divergent ECM-related features and limitations of spontaneous canine mammary tumors as comparative models; and (iv) ECM-centered omics, AI-based analysis, biomarker discovery, patient stratification, and treatment-response prediction. When available, studies directly addressing human breast cancer or canine mammary tumors were prioritized over evidence from unrelated tumor types.

Studies were excluded when they were unrelated to ECM biology or breast cancer progression; focused exclusively on non-mammary tumor models without a clear mechanistic connection to ECM remodeling; lacked sufficient methodological detail; or did not contribute directly to the conceptual scope of this review. Literature on trophoblast invasion, bovine stromal resistance, melanoma invasion, and drug–stem cell therapy was not retained as a major narrative axis in the revised manuscript because these topics were considered peripheral to the ECM-centered breast cancer and comparative oncology framework.

## 3. ECM Remodeling and Molecular Architecture as Mechanobiological Determinants of Breast Cancer Aggressiveness

### 3.1. ECM-Defined Stromal Permissiveness and Tumor Invasion

The extracellular matrix (ECM) is not a static barrier surrounding malignant cells but a dynamic, functionally adaptable compartment of the tumor microenvironment. In homeostatic mammary tissue, basement membrane integrity, balanced matrix turnover, organized collagen architecture, and controlled protease activity contribute to epithelial polarity and tissue compartmentalization. During breast cancer progression, however, this regulatory function is progressively disrupted. Tumor cells, cancer-associated fibroblasts (CAFs), endothelial cells, immune cells, and ECM-remodeling enzymes collectively reshape the matrix, converting a tissue-restraining scaffold into a permissive stromal interface that supports invasion, angiogenesis, immune modulation, and metastatic dissemination [2,3,11,30].

This transition is central to the concept of ECM-defined stromal permissiveness. Rather than depending exclusively on the intrinsic invasive capacity of tumor cells, local invasion emerges from reciprocal interactions between malignant cells and a remodeled stromal matrix. Basement membrane degradation allows carcinoma cells to breach epithelial boundaries, while increased deposition of fibrillar collagens, fibronectin accumulation, proteoglycan remodeling, and matricellular protein expression modify the biochemical and biomechanical properties of the surrounding tissue [2,3,8,11]. At the same time, matrix metalloproteinases (MMPs), heparanase, lysyl oxidase (LOX), and related remodeling enzymes generate invasion tracks, release matrix-bound growth factors, and create gradients of stiffness and ligand availability that guide tumor cell migration [3,9,11,30].

In breast cancer, stromal permissiveness is therefore best understood as a tissue-level property generated by ECM composition, architecture, mechanics, and proteolytic remodeling. Collagen fiber alignment at the tumor–stroma interface can provide directional migration paths, whereas LOX-mediated crosslinking increases matrix stiffness and amplifies integrin-dependent mechanotransduction [8,9,10]. Fibronectin-rich matrices further support focal adhesion assembly and cooperate with collagen remodeling to enhance cell contractility and invasive behavior [2,3,11]. These matrix alterations do not merely accompany tumor progression; they actively condition the physical and biochemical landscape through which tumor cells move.

Importantly, ECM-defined permissiveness also involves stromal and immune compartments. CAFs are major producers and remodelers of tumor ECM, contributing to collagen deposition, matrix contraction, MMP secretion, reciprocal tumor–stroma signaling, immune crosstalk, and the generation of mechanically heterogeneous stromal regions [3,11,31]. Immune cells can further reinforce or restrain ECM remodeling by secreting proteases, cytokines, and growth factors that regulate fibroblast activation, angiogenesis, and inflammatory signaling. Consequently, ECM remodeling links invasion to broader tumor microenvironmental programs, including immune exclusion, vascular remodeling, metabolic adaptation, and treatment resistance [3,11,31].

This framework avoids reducing invasion to a purely cell-autonomous process. Instead, it positions breast cancer aggressiveness as the result of a reciprocal and spatially organized tumor–stroma–ECM system. In this context, the ECM functions as both a substrate for invasion and a regulator of malignant phenotypes, integrating structural disruption, biochemical signaling, and biomechanical force transmission. The following subsection expands this mechanistic axis by examining how matrix stiffness, collagen architecture, integrin signaling, focal adhesion dynamics, and YAP/TAZ-dependent mechanotransduction convert ECM remodeling into intracellular programs that promote tumor aggressiveness.

### 3.2. Matrix Stiffness, Collagen Architecture, and Mechanotransduction

Matrix stiffening is one of the most functionally relevant consequences of ECM remodeling in breast cancer. In normal mammary tissue, collagen organization, basement membrane integrity, and balanced matrix turnover help preserve epithelial polarity and tissue compliance. During tumor progression, increased deposition of fibrillar collagens, LOX-mediated collagen crosslinking, fibronectin accumulation, and proteolytic matrix remodeling progressively alter tissue mechanics and generate a stiffer tumor-associated ECM [8,9,10,11,32]. This mechanical transition is not merely a passive consequence of stromal expansion. Instead, it creates a feed-forward system in which increased matrix stiffness promotes tumor cell contractility, focal adhesion maturation, cytoskeletal tension, and mechanosensitive signaling.

Collagen architecture is particularly important in this process. Fibrillar collagen deposition and alignment at the tumor–stroma interface can generate directional tracks that facilitate collective and single-cell invasion [8,10,30]. Tumor-associated collagen signatures, including increased fiber density, linearization, and perpendicular alignment relative to the tumor boundary, have been associated with invasive behavior and worse clinical outcomes in breast cancer [8,10]. LOX and LOX-like enzymes further reinforce this phenotype by catalyzing collagen crosslinking, thereby increasing matrix stiffness and enhancing integrin clustering, focal adhesion assembly, and downstream mechanotransduction [9,11]. Thus, collagen remodeling links tissue-level architecture to cell-level invasive programs.

Tumor cells sense these mechanical and structural changes primarily through integrins and focal adhesion complexes. Integrin engagement with collagen- and fibronectin-rich matrices activates focal adhesion kinase (FAK), Src-family kinases, RhoA–ROCK signaling, actomyosin contractility, and cytoskeletal tension [9,11,12,32,33]. These pathways transmit mechanical information from the extracellular environment to the nucleus, where mechanosensitive transcriptional regulators such as YAP and TAZ promote gene expression programs associated with proliferation, survival, stemness, epithelial–mesenchymal transition, invasion, and therapy resistance [11,13,32,33,34]. In this context, mechanotransduction provides a direct molecular route through which ECM remodeling regulates malignant cell behavior.

The relationship between ECM mechanics and tumor progression is reciprocal. Stiffer matrices can stimulate tumor cells and CAFs to produce additional ECM components, secrete MMPs, increase contractile force, and reinforce stromal remodeling [3,11,32,33]. CAF-mediated matrix contraction further amplifies tissue tension and spatial heterogeneity, generating regions with distinct stiffness, porosity, and ligand availability. These mechanically heterogeneous niches can influence not only invasion but also vascular compression, immune cell infiltration, hypoxia, nutrient gradients, and therapeutic penetration [3,11,32]. Therefore, matrix stiffness should be understood as an integrated property of ECM composition, architecture, cellular contractility, and enzymatic remodeling.

**Figure 1:**
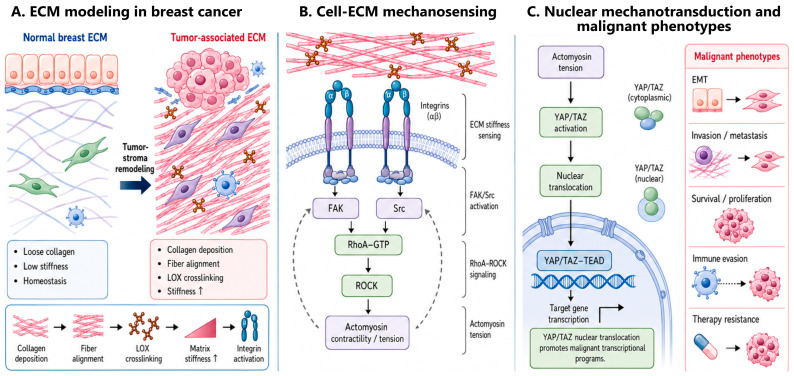
ECM remodeling and mechanotransduction in breast cancer aggressiveness. (**A**) Normal and tumor-associated breast ECM are compared, highlighting collagen deposition, fiber alignment, LOX-mediated crosslinking, and increased stiffness. (**B**) Increased ECM stiffness promotes integrin-dependent FAK/Src and RhoA–ROCK signaling, actomyosin contractility, and cytoskeletal tension. (**C**) These mechanical signals support YAP/TAZ activation and transcriptional programs associated with epithelial–mesenchymal transition, invasion/metastasis, survival/proliferation, immune evasion, and therapy resistance. This figure is intended as a conceptual synthesis of ECM mechanobiology rather than a deterministic pathway map.

Importantly, mechanotransduction is context-dependent. Increased stiffness or collagen deposition does not produce uniform biological effects across all tumors because stiffness-driven signaling depends on tumor lineage, stromal composition, immune contexture, mechanosensor expression, matrix topology, protease activity, spatial organization, and therapeutic pressure [7,32]. For this reason, ECM mechanics should not be interpreted as a single biomarker in isolation, but as part of a broader matrix-defined phenotype that integrates collagen architecture, focal adhesion signaling, CAF activation, immune modulation, and treatment response. The transition from a homeostatic mammary ECM to a tumor-associated, mechanically permissive matrix involves coordinated changes in collagen deposition and alignment, basement membrane integrity, matrix crosslinking, integrin-mediated mechanosensing, focal adhesion signaling, and YAP/TAZ-dependent transcriptional regulation (Figure 1).

### 3.3. Structural Proteins, Proteoglycans, Matricellular Regulators, and ECM-Remodeling Enzymes

The tumor ECM is composed of structurally and functionally diverse molecular classes that collectively regulate tissue architecture, biochemical signaling, and biomechanical behavior. Rather than acting as isolated biomarkers, ECM components operate as an interconnected matrix network in which structural proteins, proteoglycans, glycosaminoglycans, matricellular proteins, and remodeling enzymes cooperate to shape tumor aggressiveness [4,11,14,16,24]. This molecular organization is dynamic and context-dependent, varying according to tumor subtype, stromal composition, spatial localization, enzymatic activity, and interactions with immune and vascular compartments.

Structural ECM proteins provide the architectural foundation for tumor–stroma organization. Collagens, particularly fibrillar collagens I and III and basement membrane collagen IV, regulate stiffness, tissue compartmentalization, and invasion routes. Increased collagen deposition, fiber alignment, and crosslinking contribute to mechanical reinforcement of the tumor microenvironment, whereas basement membrane disruption facilitates epithelial boundary breach and stromal invasion [2,8,9,10,11]. Fibronectin promotes integrin-dependent adhesion, focal adhesion assembly, and invasive migration, while laminins regulate epithelial polarity, basement membrane organization, and angiogenic signaling. Elastin and elastin-derived fragments may further influence cell migration, vascular remodeling, and protease activity. Together, these structural proteins define both the physical scaffold and the mechanobiological interface through which tumor cells interact with the surrounding stroma [2,4,11].

Proteoglycans and glycosaminoglycan-associated molecules add a regulatory layer by controlling matrix hydration, growth-factor availability, receptor engagement, and inflammatory signaling. Molecules such as decorin, biglycan, lumican, versican, perlecan, glypicans, and CSPG4 can exert tumor-suppressive or tumor-promoting effects depending on cellular context and matrix state [2,11,35]. Decorin and lumican, for example, may restrain tumor progression by modulating collagen fibrillogenesis, TGF-β signaling, and growth-factor receptor activity, whereas biglycan, versican, perlecan, and CSPG4 are frequently associated with inflammation, angiogenesis, fibroblast activation, migration, and invasion. These dual and sometimes opposing effects emphasize that proteoglycans should not be interpreted as uniformly pro- or anti-tumoral, but rather as context-dependent regulators of ECM organization and tumor microenvironmental signaling.

Matricellular proteins function primarily as signaling modulators rather than structural scaffolds. Tenascin-C, periostin, osteopontin, SPARC, and thrombospondins regulate cell–matrix adhesion, integrin signaling, CAF activation, angiogenesis, immune modulation, collagen remodeling, and metastatic niche formation [14,15]. Their biological effects are highly dependent on tumor stage, stromal localization, receptor availability, and mechanical context. Periostin and tenascin-C can cooperate with collagen and fibronectin networks to support invasive niches and metastatic colonization, whereas SPARC and thrombospondins may exhibit either tumor-restraining or tumor-promoting functions depending on the microenvironment. This functional plasticity reinforces the need to interpret matricellular proteins as dynamic regulators of tumor–ECM crosstalk rather than as linear markers of aggressiveness.

**Figure 2:**
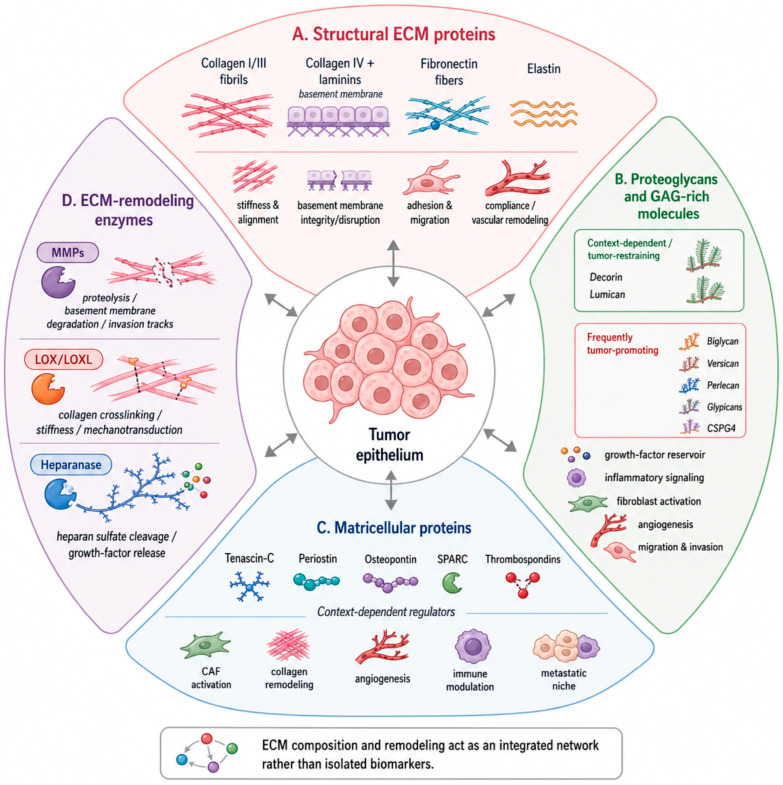
Major ECM molecular and functional classes involved in breast cancer aggressiveness. The tumor-associated ECM is represented as an integrated network of structural ECM proteins, proteoglycans and GAG-associated molecules, matricellular proteins, and ECM-remodeling enzymes interacting with tumor epithelium. These classes regulate stiffness, basement membrane integrity, adhesion, migration, CAF activation, immune modulation, angiogenesis, proteolysis, collagen crosslinking, mechanotransduction, and growth-factor release. This figure is intended as a conceptual synthesis of ECM network organization in breast cancer rather than a deterministic linear pathway.

ECM-remodeling enzymes provide the catalytic machinery that converts ECM composition into biological activity. Matrix metalloproteinases (MMPs), LOX/LOXL enzymes, heparanase, and related proteases remodel the ECM by degrading basement membrane components, generating invasion tracks, releasing matrix-bound growth factors, producing bioactive ECM fragments, and modifying collagen crosslinking [3,9,11,30,36]. Through these mechanisms, ECM-remodeling enzymes regulate not only invasion but also angiogenesis, immune infiltration, vascular permeability, tissue stiffness, and therapeutic penetration. Therefore, the molecular architecture of the tumor ECM should be viewed as an integrated regulatory system in which matrix composition, ECM-remodeling enzymes, cellular contractility, and spatial organization collectively determine whether the stroma restrains or facilitates malignant progression. Key ECM components, ECM-remodeling enzymes, and their major functional outputs related to breast cancer aggressiveness are summarized in Table 1, following a matrisome-informed functional organization, whereas their network-level interactions are illustrated conceptually in Figure 2 [14,16,24].

## 4. Spontaneous Canine Mammary Tumors in Comparative Oncology: Conserved ECM Mechanisms and Translational Constraints

### 4.1. Biological Rationale for Spontaneous Canine Mammary Tumors in ECM-Centered Comparative Oncology

Spontaneous canine mammary tumors provide a relevant comparative framework for investigating ECM-driven tumor progression because they arise naturally in immunocompetent hosts and develop within an intact tumor–stroma–ECM microenvironment. Unlike transplant-based, chemically induced, or highly reductionist experimental models, spontaneous tumors evolve under endogenous hormonal, inflammatory, stromal, vascular, and mechanical influences. This is particularly important for ECM-centered oncology because matrix remodeling is not an isolated tumor-cell process, but a tissue-level phenomenon shaped by cancer-associated fibroblasts, immune cells, endothelial cells, proteases, collagen remodeling, matrix stiffness, and spatially organized tumor–stroma interactions [17,18,19,20,22].

The comparative relevance of canine mammary tumors should therefore be understood primarily in terms of biological context. These tumors may preserve aspects of tumor–matrix co-evolution that are difficult to reproduce in vitro or in xenograft models, including progressive stromal remodeling, heterogeneous collagen organization, variable immune infiltration, angiogenic adaptation, and interactions between neoplastic cells and native mammary stroma [17,18,19,20,21,22]. For ECM-focused studies, this spontaneous setting offers an opportunity to examine how matrix composition, architecture, and remodeling enzymes participate in invasion, stromal permissiveness, vascular remodeling, and metastatic behavior under naturally occurring disease conditions.

However, the comparative value of canine mammary tumors does not imply direct equivalence to human breast cancer. Important interspecies differences exist in molecular subtype distribution, hormone receptor status, reproductive history, age, breed-related variability, clinical management, treatment exposure, tissue sampling, fixation protocols, and histopathological classification systems [17,18,19,20,22]. These factors can influence ECM composition, stromal organization, immunohistochemical profiles, and interpretation of prognostic markers. Therefore, canine mammary tumors are best positioned as complementary spontaneous models for identifying conserved and divergent ECM-related mechanisms, rather than as direct replicas of all human breast cancer subtypes.

This distinction is essential for translational interpretation. When carefully integrated with human breast cancer datasets, standardized pathology, ECM-focused imaging, proteomics, spatial analysis, and clinical metadata, canine mammary tumors may contribute to hypothesis generation and cross-species validation of ECM-associated signatures [21,22,23,24,37,38,39]. In this context, canine mammary tumors may be particularly useful for clarifying whether specific matrix features—such as collagen remodeling, fibronectin accumulation, MMP activity, CAF-associated stromal expansion, and mechanotransduction-related markers—represent broadly conserved stromal programs or species- and context-dependent phenomena. In this framework, comparative oncology becomes a bridge between mechanistic ECM biology and translational biomarker discovery, while still requiring rigorous attention to model-specific constraints.

### 4.2. Cross-Species Conservation of ECM-Driven Stromal Remodeling

Cross-species conservation in ECM-driven stromal remodeling should be interpreted as the recurrence of comparable stromal programs rather than as complete molecular equivalence between human breast cancer and canine mammary tumors. In both species, tumor progression involves reciprocal interactions among malignant epithelial cells, cancer-associated fibroblasts or cancer-associated stroma, immune and vascular compartments, matrix-remodeling enzymes, and ECM structural components [2,3,11,17,18,19,20,22]. These interactions can generate convergent microenvironmental states in which stromal activation, matrix remodeling, protease activity, angiogenic adaptation, immune modulation, and mechanotransduction-related signaling collectively support invasive and metastatic behavior.

Stromal transcriptomic and proteomic profiling provides more direct support for this interpretation. Gene-expression analyses of cancer-associated stroma from canine mammary tumors have identified molecular similarities with human breast carcinomas, indicating that selected stromal remodeling programs are at least partially conserved across species [37,40]. Subsequent studies using laser-capture microdissection and FFPE-compatible transcriptomic or proteomic approaches further showed that canine mammary tumors display disease-associated stromal reprogramming involving ECM organization, fibroblast activation, immune-related pathways, and tumor-promoting stromal components [23,40,41,42]. These findings are particularly relevant for ECM-centered comparative oncology because they indicate that the canine tumor stroma can preserve biologically meaningful remodeling programs beyond tumor-cell-intrinsic alterations.

Rather than representing isolated molecular overlaps, these conserved stromal programs appear to involve coordinated functional modules. CAF-associated ECM deposition, MMP-related matrix degradation, TGF-β-linked fibroblast activation, immune-cell recruitment, fibronectin-rich adhesive signaling, and collagen-associated remodeling can converge to generate permissive stromal niches in both human and canine mammary tumors [2,3,11,23,37,40,41,42]. Within these niches, matrix remodeling may influence immune infiltration and vascular organization, whereas activated fibroblasts can reinforce ECM deposition, contractility, proteolysis, and mechanotransduction. This reciprocal organization supports the concept that ECM-driven stromal remodeling can act as a conserved tissue-level program linking tumor architecture to aggressive behavior.

Accordingly, canine mammary tumors should be used as complementary spontaneous models for testing which stromal remodeling modules are conserved, which are species- or context-dependent, and which require validation in matched human datasets. Conservation is expected to be partial and shaped by histological subtype, hormone receptor status, reproductive history, breed-related variability, treatment exposure, tissue processing, and analytical methodology [17,18,19,20,22]. For this reason, cross-species ECM comparisons should prioritize standardized pathology, spatially resolved stromal assessment, ECM-focused imaging, and matched molecular profiling. This framework provides the basis for evaluating whether matrix-derived features are merely descriptive tissue alterations or biologically informative readouts of tumor aggressiveness.

### 4.3. ECM Architecture and Spatial Organization as Prognostic Features in Canine Mammary Tumors

ECM architecture in canine mammary tumors should be interpreted as a spatially organized tissue phenotype rather than as a static histological background. In this context, matrix architecture includes collagen fiber density, width, length, straightness, alignment, tumor–stromal boundary definition, basement membrane continuity, stromal cellularity, and the spatial relationship between neoplastic epithelial cells and surrounding matrix compartments. These features are relevant because they reflect how tumor cells interact with the native mammary stroma, remodel the extracellular matrix, and generate permissive niches for local invasion and metastatic dissemination [2,3,11,21,43,44]. However, ECM architecture should complement, not replace, established histopathological classification, tumor grading, clinical staging, and assessment of lymphovascular invasion [17,18,19,20,22,45].

Among ECM-derived architectural features, collagen organization currently provides a relevant quantitative evidence base in canine mammary tumors. Second harmonic generation (SHG) imaging has been used to evaluate fibrillar collagen density, tumor-associated collagen signatures, tumor–stromal boundary definition, and individual collagen fiber parameters in canine mammary carcinoma samples [43,44]. These studies indicate that collagen architecture is not merely a descriptive stromal feature. Instead, specific collagen parameters, including fiber width, fiber length, fiber straightness, and tumor–stromal boundary organization, have been associated with adverse clinicopathological behavior and survival outcomes in dogs with mammary carcinoma [21,43]. These findings support the interpretation of collagen architecture as a candidate prognostic layer within the canine mammary tumor microenvironment.

The prognostic relevance of collagen architecture is also supported by studies integrating morphological and molecular collagen features. Quantitative analyses of canine mammary cancer have associated collagen fiber characteristics with tumor behavior and survival time, while molecular phenotyping of type I collagen has shown that neoplastic canine mammary tissues can exhibit altered collagen fiber morphology and post-translational collagen modifications compared with non-neoplastic mammary tissue [21,46]. More recent analyses further suggest that collagen modifications may help discriminate biologically distinct tumor regions and may be associated with lymph node metastasis in dogs with carcinoma in mixed tumors [47]. Comparative evaluation of collagen modifications in human and canine mammary carcinomas also supports the presence of shared matrix alterations across species, although these features should be interpreted as context-dependent rather than universally conserved [39].

Importantly, matrix architecture extends beyond collagen alone. Basement membrane disruption, fibronectin-rich adhesive interfaces, stromal expansion, protease-associated remodeling, immune-cell infiltration, and vascular remodeling can all influence how ECM-derived features relate to aggressiveness. In canine mammary tumors, these variables may differ according to histological subtype, tumor grade, reproductive history, hormone receptor status, stromal composition, sampling site, and tissue processing [17,18,19,20,22,45]. Therefore, spatial organization should be evaluated within pathologist-guided regions of interest that distinguish intratumoral stroma, invasive fronts, peritumoral matrix, necrotic areas, and epithelial-rich versus stromal-rich compartments. Without this spatial control, quantitative ECM features may reflect sampling bias rather than biologically meaningful tumor behavior.

For translational interpretation, ECM architecture should therefore be considered a candidate prognostic readout that should be integrated with clinicopathological and molecular data. SHG, multiphoton microscopy, Picrosirius red polarization, immunohistochemistry, and digital pathology can provide complementary information on collagen organization, matrix distribution, basement membrane integrity, and stromal remodeling. Nevertheless, these approaches require standardized tissue handling, image acquisition, segmentation parameters, region-of-interest (ROI) selection, and statistical models that account for tumor grade, stage, lymphovascular invasion, treatment history, and molecular subtype [43,44,45,46,47]. In this framework, ECM architecture becomes most informative when interpreted as part of a spatially resolved tumor–stroma phenotype, rather than as a standalone biomarker. The next subsection addresses the translational constraints that must be considered before ECM-derived features can be used reliably for biomarker discovery and cross-species validation.

### 4.4. Translational Constraints and ECM-Based Biomarker Discovery

At this stage, the central issue is not whether ECM alterations are biologically relevant but whether they can be converted into reliable biomarker platforms. In canine mammary tumors, this requires distinguishing descriptive ECM-associated findings from biomarkers supported by analytical validity, biological plausibility, clinical validity, and clinical utility [21,23,24,37,38,39,40,41,42,43,44,45,46,47,48,49]. Without this evidentiary hierarchy, cross-species ECM observations may remain mechanistically informative but insufficient for prognosis, patient stratification, or treatment-response prediction.

The first major constraint is heterogeneity in case definition. Canine mammary tumors include diverse histological subtypes and grades, and these categories are not interchangeable for ECM biomarker analysis. Mixed tumors, complex carcinoma, solid carcinoma, tubular carcinoma, inflammatory carcinoma, and metastatic lesions may differ substantially in stromal abundance, collagen organization, epithelial–stromal ratio, inflammatory infiltrate, and invasive pattern [17,18,19,20,22,45,50]. In addition, reproductive history, ovariohysterectomy status, age, breed, hormone receptor status, clinical stage, lymphovascular invasion, lymph node involvement, prior treatment, and follow-up duration can all influence how ECM-derived features relate to outcome. Therefore, ECM biomarker studies should define the target population, tumor subtype, grade, stage, nodal status, treatment history, and outcome endpoint before testing matrix-derived signatures.

The second constraint is the frequent dependence on retrospective veterinary cohorts. Such cohorts are valuable, but they often include heterogeneous fixation times, ischemic intervals, tissue block selection criteria, archival duration, clinical follow-up, treatment histories, and metadata completeness. These variables can affect both molecular measurements and histological interpretation while also limiting the ability to adjust for confounders. In this setting, apparent ECM-associated prognostic effects may reflect cohort composition, tissue preservation, or incomplete clinical annotation rather than reproducible tumor biology. This is particularly relevant when studies combine benign and malignant lesions, primary and metastatic samples, treated and untreated cases, or tumors with different histological grades within the same analysis [17,18,19,20,22,43,44,45,46,47,50].

The third constraint is assay transferability across species and laboratories. Biomarker studies based on immunohistochemistry, molecular profiling, or image-derived matrix features require evidence that the assay performs consistently in canine tissue and remains robust across processing conditions. Antibody specificity, antigen preservation, scoring systems, observer variability, platform differences, and site-specific analytical choices can all affect whether a candidate ECM marker is reproducible. Therefore, reported associations should be interpreted cautiously unless they are supported by appropriate controls, transparent reporting of pre-analytical variables, reproducible scoring or feature-extraction procedures, and independent confirmation [43,44,45,46,47,48,49,50].

Validation is the central requirement for moving from ECM-associated findings to biomarker platforms. Candidate ECM signatures should ideally be tested across independent canine cohorts, human breast cancer datasets, and, when appropriate, cross-species comparative analyses. Predictive models should be evaluated for calibration, discrimination, reproducibility, external validity, interpretability, and robustness to confounders such as tumor subtype, grade, stage, treatment exposure, and site of tissue collection [27,28,29,48,49,51]. In this framework, multi-omics integration and AI-assisted analysis should be introduced only after robust case definition, assay reproducibility, and validation strategy have been established.

**Figure 3:**
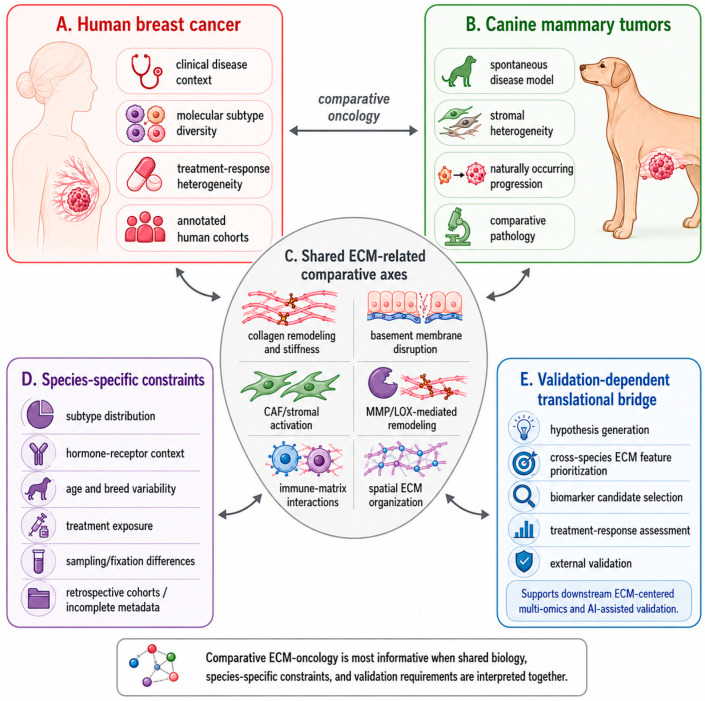
Comparative ECM–oncology framework linking human breast cancer and spontaneous canine mammary tumors. Human breast cancer provides clinically annotated disease contexts with molecular subtype diversity, treatment-response heterogeneity, and patient-derived datasets, whereas canine mammary tumors provide a complementary spontaneous model that may preserve tumor–stroma–ECM interactions under intact host conditions. Shared comparative axes include collagen remodeling, matrix stiffness, basement membrane disruption, CAF/stromal activation, MMP/LOX-mediated remodeling, immune–matrix interactions, and spatial ECM organization. Species-specific constraints, including differences in subtype distribution, hormone-receptor context, age and breed variability, treatment exposure, tissue processing, retrospective cohort design, and metadata completeness, must be considered before cross-species extrapolation. This figure is intended as a conceptual comparative framework rather than a direct equivalence model between human and canine mammary tumors.

Under these conditions, canine mammary tumors can support hypothesis generation and cross-species prioritization of ECM-associated signatures, providing a disciplined foundation for the multi-omics and AI-based predictive platforms discussed in the next section (Figure 3).

## 5. Translational Tools: ECM-Centered Multi-Omics, Artificial Intelligence, and Predictive Biomarker Platforms

The translational use of ECM-centered data depends on the ability to connect matrix biology, spatial tissue organization, molecular profiling, computational modeling, and clinically meaningful endpoints. As discussed above, ECM-derived alterations should not be assumed to function as biomarkers solely because they are biologically associated with tumor aggressiveness. Their utility depends on whether they can be measured reproducibly, interpreted within defined biological and clinicopathological contexts, validated across cohorts, and integrated into models that support prognosis, patient stratification, or treatment-response assessment. In this section, we discuss multi-omics, spatial matrix profiling, artificial intelligence, predictive biomarker platforms, and ECM-targeted therapeutic strategies as translational tools whose value depends on analytical rigor, biological plausibility, external validation, and clinical interpretability.

### 5.1. ECM-Centered Multi-Omics and Spatial Matrix Profiling

ECM-centered multi-omics should be understood not only as a strategy for describing tumor composition but also as a way to test which matrix-associated programs are conserved, divergent, spatially localized, metabolically coupled, and clinically meaningful across human breast cancer and spontaneous canine mammary tumors. This distinction is important because the comparative value of canine mammary tumors depends on identifying reproducible stromal programs rather than assuming direct equivalence between species. In this framework, matrix-enriched proteomics, matrisome-informed annotation, transcriptomic profiling, lipidomics, metabolomics, spatial omics, mass spectrometry imaging, and histopathology can provide complementary layers of evidence when integrated with tumor subtype, grade, tissue compartment, treatment history, and outcome data [14,16,23,24,25,40,41,42,52,53].

Matrisome-informed profiling is particularly useful because it shifts the analysis from isolated candidate markers to coordinated ECM programs. By distinguishing core structural ECM components from ECM-associated glycoproteins, proteoglycans, ECM-affiliated molecules, and remodeling regulators, the matrisome framework enables cross-species comparisons that focus on functional matrix modules rather than single molecules [14,16,24,52]. In a comparative oncology context, this approach can be used to ask whether stromal programs described in human breast cancer, such as collagen-rich matrix remodeling, basement membrane alteration, fibronectin-associated adhesion, matricellular signaling, protease-associated matrix turnover, and CAF-linked ECM deposition, also emerge in canine mammary tumors after controlling for histological subtype, grade, tissue compartment, and clinical annotation [23,40,41,42,52,53]. Such comparisons are stronger when they evaluate conserved pathways or matrix modules, rather than isolated abundance changes that may reflect species-specific biology, sampling bias, or cohort composition.

Proteomics and ECM-focused protein profiling are central to this framework because ECM proteins are not always accurately inferred from transcript abundance. Matrix proteins differ in solubility, extractability, crosslinking state, degradation, post-translational modification, and spatial distribution, all of which can influence their detection and biological interpretation [14,23,24,52]. Matrix-enriched proteomics can therefore help determine whether aggressive tumors show coordinated enrichment of structural ECM proteins, matricellular regulators, proteases, crosslinking enzymes, or stromal activation signatures. In canine mammary tumors, stromal transcriptomic and proteomic studies have suggested disease-associated remodeling involving ECM organization, fibroblast activation, immune-related pathways, and tumor-promoting stromal components [23,40,41,42]. In addition, ECM protein-pattern analysis in canine neoplastic mammary glands has provided direct evidence that tumorigenesis is associated with alterations in the mammary ECM protein microenvironment [53]. The translational question is whether these canine stromal and ECM protein signatures align with matrix programs observed in human breast cancer and whether they retain prognostic or treatment-response relevance after adjustment for tumor heterogeneity and methodological variability.

Lipidomics and metabolomics add a functional layer by linking ECM remodeling to metabolic adaptation. In cancer, metabolic reprogramming is shaped not only by malignant epithelial cells but also by stromal composition, nutrient availability, hypoxia, oxidative stress, immune contexture, and therapy pressure [54,55,56,57,58,59,60]. In breast cancer specifically, altered lipid metabolism has been associated with tumor progression and may provide information on membrane composition, bioenergetic adaptation, signaling lipids, and treatment-relevant metabolic states [55,61]. These interactions are relevant to ECM-centered oncology because matrix stiffness, collagen density, hypoxia, CAF activity, and tumor–stroma crosstalk can reshape the biochemical environment in which malignant cells, fibroblasts, immune cells, and endothelial cells compete for nutrients and adapt to stress [54,55,56,57,58,59,60,61]. In canine mammary tumors, however, lipidomic and metabolomic evidence remains less mature than histological, transcriptomic, or proteomic evidence. Therefore, these approaches should be framed as emerging tools for testing whether ECM-rich or mechanically remodeled tumor regions exhibit comparable metabolic states across species, rather than as established comparative biomarkers.

Spatial lipidomics and metabolomics may be particularly relevant to this question, as metabolic signatures can vary substantially across tumor compartments. Although direct evidence linking spatial lipidomic or metabolomic profiles to ECM-defined canine mammary tumor biomarkers remains limited, mass spectrometry imaging approaches, such as DESI-MS, have already been applied to map lipid and metabolite distributions in mammalian tissues and animal models while preserving morphological integrity [62,63]. These studies support the technical feasibility of spatial molecular profiling in comparative animal models, even though their application to ECM-centered canine mammary tumor biomarker discovery remains to be established. In future comparative studies, spatial lipid and metabolite imaging could help determine whether collagen-rich stroma, invasive fronts, hypoxic regions, immune-enriched compartments, or metastatic niches are associated with specific metabolic states. Such analyses will require careful control of tumor subtype, sampling region, ischemic time, storage conditions, extraction method, systemic metabolic variables, and treatment exposure.

Single-cell and spatially resolved breast cancer studies provide an important human reference layer for ECM-centered interpretation, showing that breast tumors are organized into heterogeneous epithelial, stromal, immune, vascular, and spatially defined states rather than homogeneous molecular entities [64,65]. For ECM-centered analyses, this is relevant because matrix-associated signals may differ across tumor compartments, stromal neighborhoods, immune organization, invasive-front localization, and molecular subtypes. However, these human spatial datasets should be used to guide compartment-aware hypotheses and feature prioritization rather than as direct evidence that equivalent spatial states are conserved in canine mammary tumors.

A further limitation is that ECM-centered multi-omics depends on interoperability across laboratories, platforms, and data types. Differences in tissue sampling, ischemic time, fixation, storage, matrix-enrichment protocols, protein or metabolite extraction, spatial-omics platforms, mass spectrometry instrumentation, image acquisition, annotation procedures, and metadata completeness can introduce substantial variability before computational integration begins [25,27,28,29,66,67,68]. Therefore, harmonized reporting, open-standard data formats, shared metadata structures, transparent preprocessing pipelines, and cross-site quality-control procedures are essential for comparing or combining ECM-related datasets. These requirements extend beyond technical standardization. Data-sharing policies, institutional governance, infrastructure costs, intellectual-property constraints, and unequal access to high-throughput platforms can limit the formation of sufficiently large and diverse multicenter cohorts. Consequently, the development of generalizable ECM-centered predictive models depends not only on analytical performance but also on coordinated data governance and sustainable collaborative infrastructures.

For comparative oncology, the most informative multi-omics strategy is therefore not the accumulation of independent molecular layers but their integration into a controlled translational framework. Candidate ECM signatures should be evaluated according to whether they are reproducible across cohorts, traceable to defined tissue compartments, compatible with known matrix biology, robust to species-specific differences, and associated with clinically meaningful endpoints. Multi-omics and spatial profiling can thus refine the selection of ECM-associated biomarker candidates, but they do not eliminate the need for standardized pathology, harmonized metadata, external validation, and cautious interpretation of cross-species similarity.

### 5.2. AI-Assisted Integration and Predictive Modeling of ECM Signatures

Artificial intelligence (AI) and machine-learning approaches may help integrate ECM-centered imaging, molecular profiling, spatial omics, and clinicopathological data, but their translational value depends on the quality, biological relevance, and validation of the input data. In ECM-centered oncology, AI should not be viewed as an independent solution to biological complexity. Rather, it should be used as an analytical framework to test whether matrix-associated features can improve the prediction of clinically meaningful outcomes when combined with pathology, molecular data, spatial information, and clinical metadata [26,27,28,29,51,69,70,71,72,73,74].

Digital pathology is one of the most immediate entry points for AI-assisted ECM analysis because it can operationalize matrix features that are difficult to capture consistently by visual assessment alone. Rather than merely quantifying previously described histological patterns, AI-based approaches can test whether stromal architecture, epithelial–stromal organization, collagen-associated features, immune–matrix relationships, and invasive-front morphology add prognostic or predictive information beyond established clinicopathological variables [43,44,45,46,47,51,66,67,68,69,70,71]. In this context, the key translational question is not whether computational models can detect ECM-related patterns, but whether these patterns are reproducible, biologically interpretable, and independently informative across cohorts, platforms, and species. This question is particularly important because ECM-derived computational features are shaped by both biological variation and technical factors introduced during tissue processing, image acquisition, annotation, and feature extraction.

These dependencies pose a major challenge to model generalization, as ECM-related image features are highly sensitive to tissue preparation, staining variability, scanner differences, region selection, segmentation strategy, and cohort composition. Algorithms trained on one institution, staining protocol, scanner platform, species, or tumor subtype may not generalize to independent datasets [28,29,69,70,71,72,73,74]. This is particularly relevant for comparative oncology because canine mammary tumors differ from human breast cancer in histological classification, subtype distribution, tissue processing, clinical management, and outcome annotation. Therefore, AI models trained to detect or quantify ECM-associated signatures should be evaluated across independent cohorts and, when possible, across laboratories, scanners, tissue-processing workflows, and species-specific pathology settings.

AI-assisted integration may be especially useful when ECM information is multimodal. For example, collagen architecture, matrisome-derived protein signatures, stromal transcriptomic programs, spatial immune organization, lipid metabolic states, and clinical variables may each provide partial information about tumor aggressiveness. Machine-learning models can be used to test whether these layers improve risk stratification, metastasis prediction, immune-exclusion assessment, or treatment-response estimation compared with conventional clinicopathological models alone [25,26,27,28,29,48,49,51,69,70,71,72,73,74]. Nevertheless, multimodal integration also increases the risk of overfitting, data leakage, batch effects, hidden confounding, and non-reproducible feature selection. These risks are particularly high in small retrospective veterinary cohorts, where sample size, metadata completeness, treatment heterogeneity, and follow-up duration may limit model robustness.

Model evaluation should therefore be explicitly validation-oriented. Performance should not be reported solely by apparent accuracy on the training dataset. Predictive models should be assessed using appropriate internal validation, preferably followed by external validation in independent cohorts. Calibration, discrimination, sensitivity, specificity, positive and negative predictive values, confidence intervals, decision-curve analysis when appropriate, and clinically meaningful endpoints should be reported transparently [28,29,48,49,72,73,74]. For ECM-centered models, it is also important to test whether matrix-derived features remain informative after adjustment for tumor subtype, grade, stage, nodal status, treatment exposure, and other established prognostic variables. Without this comparison, AI-derived ECM signatures may reflect known clinical or histological differences rather than independent biological information.

Explainability is another critical requirement. Because ECM features are spatially and biologically complex, black-box models may identify patterns that are statistically predictive but biologically uninterpretable or technically confounded. Explainable AI approaches, feature-importance analyses, attention maps, spatial attribution methods, and pathologist-guided reviews can help determine whether model predictions are driven by biologically plausible matrix regions, such as invasive fronts, CAF-rich stroma, collagen-dense areas, immune-excluded compartments, or vascular niches [51,69,70,71,72,73,74]. However, explainability outputs should also be interpreted cautiously, as visually compelling heatmaps do not necessarily establish causal relevance or biological validity.

In comparative oncology, the strongest use of AI is therefore not to replace pathology or molecular validation, but to prioritize ECM-associated signatures that are reproducible, spatially interpretable, biologically plausible, and externally validated. AI-assisted models may help identify candidate matrix phenotypes shared between human breast cancer and canine mammary tumors, but cross-species prediction requires particular caution. A model that performs well in one species may capture species-specific histology, processing artifacts, or cohort structure rather than conserved tumor biology. For this reason, cross-species AI should be framed primarily as a tool for hypothesis generation, feature prioritization, and validation-oriented integration, rather than as immediate clinical decision support.

### 5.3. ECM-Based Biomarker Platforms for Stratification and Treatment-Response Assessment

The transition from ECM-associated findings to biomarker platforms requires a clear distinction between biological association, prognostic value, and predictive utility. A matrix feature may be biologically linked to invasion, immune modulation, metastasis, or therapeutic resistance, but this does not automatically make it a clinically useful biomarker. Prognostic biomarkers provide information on outcome independent of treatment, whereas predictive biomarkers identify the likelihood of benefit or resistance to a specific therapeutic strategy. Therefore, ECM-based biomarker development should follow a stepwise evidentiary framework that includes analytical validity, biological plausibility, clinical validity, and clinical utility [48,49,75]. For AI-assisted or multimodal biomarker models, additional requirements include transparent reporting, appropriate model development, calibration, discrimination, external validation, and assessment of bias or applicability [28,29,72,73,74].

ECM-based biomarker platforms are unlikely to rely solely on single molecules. Because ECM remodeling is spatially organized and biologically heterogeneous, more informative platforms may combine molecular, architectural, spatial, and clinicopathological features. Examples include matrisome-derived protein modules, collagen organization, stromal abundance, basement membrane integrity, CAF-associated matrix remodeling, protease-related signatures, immune–matrix relationships, and spatially resolved tumor–stroma interfaces [14,16,23,24,43,44,45,46,47,52,53,66,67,68]. These features should be interpreted as integrated matrix phenotypes rather than isolated readouts. Their value depends on whether they improve risk classification beyond established variables such as tumor subtype, grade, stage, lymphovascular invasion, nodal status, receptor profile, treatment exposure, and outcome definition.

For patient stratification, ECM-centered biomarkers may help identify tumors with matrix-defined aggressive phenotypes, including collagen-rich stromal remodeling, invasive-front organization, CAF-dominant stromal activation, immune-excluded microenvironments, or protease-associated tissue remodeling. In human breast cancer, such matrix phenotypes may complement molecular subtype and conventional pathology by capturing stromal and mechanobiological dimensions of tumor behavior [2,3,8,9,10,11,32,33,34,51]. In canine mammary tumors, comparable ECM-associated features may support biological prioritization and cross-species validation when evaluated within well-defined histological, clinical, and treatment contexts [17,18,19,20,21,22,23,37,40,41,42,43,44,45,46,47,50,53]. However, thresholds, scoring systems, and prognostic categories should not be transferred directly between species without validation because stromal composition, histopathological classification, sample processing, and clinical management differ substantially.

Treatment-response assessment represents a more demanding level of biomarker development. ECM remodeling can influence therapeutic response through multiple mechanisms, including altered drug penetration, interstitial pressure, hypoxia, integrin-dependent survival signaling, mechanotransduction, immune exclusion, metabolic adaptation, and therapy-induced stromal remodeling [3,7,11,31,32,33,34,54,55,56,76,77,78,79]. These mechanisms support the biological plausibility of ECM-based predictive biomarkers, but they do not, by themselves, establish predictive utility. To support treatment-response prediction, candidate ECM signatures should be evaluated in cohorts with defined treatment regimens, standardized response criteria, adequate follow-up, and appropriate control for confounding variables. Without these conditions, apparent associations between ECM features and treatment outcomes may reflect tumor stage, subtype, treatment selection, or sampling bias rather than true predictive value.

A practical ECM-based biomarker platform should therefore be designed as a layered system. The first layer should establish a reproducible measurement of the ECM feature or signature. The second should demonstrate biological coherence with known mechanisms of matrix remodeling, mechanotransduction, immune modulation, or metabolic adaptation. The third should test clinical validity in independent cohorts and determine whether the ECM signature adds information beyond established clinicopathological factors. The fourth should evaluate whether the biomarker can inform a meaningful clinical or translational decision, such as risk stratification, follow-up intensity, patient selection for mechanobiology-oriented studies, or prioritization of ECM-targeted therapeutic hypotheses [48,49,75]. In comparative oncology, canine mammary tumors may contribute most strongly to the second and third layers by enabling biological prioritization and cross-species validation of matrix-associated signatures under spontaneous disease conditions.

**Figure 4:**
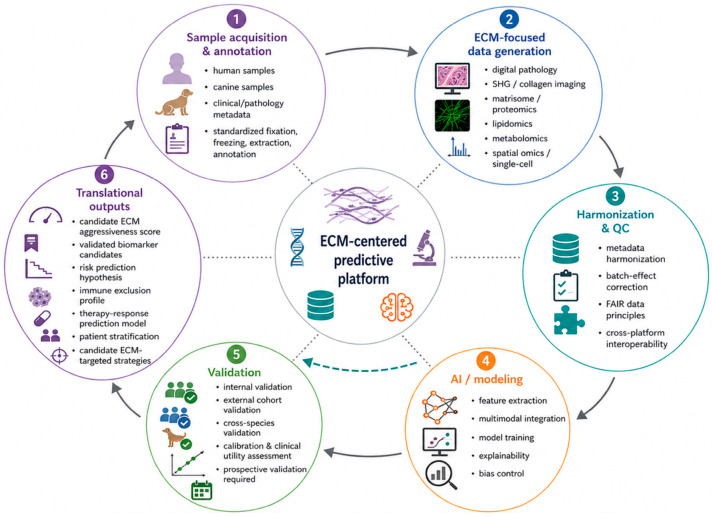
ECM-informed predictive platform for biomarker discovery, response prediction, and validation-guided translation. This conceptual workflow illustrates how human and canine samples, clinical/pathological metadata, ECM-focused molecular and spatial profiling, harmonization, quality control, AI-assisted modeling, and validation can be integrated into predictive biomarker development. Key data layers include digital pathology, SHG/collagen imaging, matrisome/proteomics, lipidomics, metabolomics, spatial omics, and single-cell approaches. Robust translation requires metadata harmonization, batch-effect control, model explainability, bias assessment, internal and external validation, calibration, clinical utility assessment, and prospective validation. Potential outputs include ECM-based aggressiveness scores, biomarker candidates, response-prediction models, patient stratification strategies, and candidate ECM-targeted therapeutic strategies. This figure is intended as a conceptual framework rather than a validated clinical pipeline.

The main translational risk is premature biomarker designation. ECM signatures derived from proteomics, imaging, spatial omics, or AI models may appear statistically robust within a discovery cohort but fail when tested across independent populations, laboratories, species, or treatment settings. For this reason, ECM-based biomarker platforms should be presented as validation-dependent frameworks rather than ready-to-use clinical tools unless supported by prospective or externally validated evidence. This cautious interpretation is particularly important for canine mammary tumors, where retrospective cohorts, heterogeneous treatments, incomplete follow-up, and variable tissue processing can limit clinical inference. Within these boundaries, ECM-centered biomarker platforms can provide a rational bridge between matrix biology, comparative oncology, and treatment-response research, while also identifying ECM-associated mechanisms suitable for therapeutic exploration. Together, these requirements support an ECM-centered predictive platform in which sample annotation, ECM-focused data generation, harmonization, AI-assisted modeling, and internal, external, cross-species, and prospective validation are integrated before translational outputs are interpreted as clinically meaningful biomarker or treatment-response tools (Figure 4).

### 5.4. ECM-Targeted Therapeutic Translation: Opportunities and Boundaries

ECM-targeted therapeutic translation represents a logical extension of ECM-centered biomarker discovery, but it should be interpreted with caution. The tumor ECM contributes to invasion, mechanotransduction, immune modulation, metabolic adaptation, drug penetration, and treatment resistance; however, these functions are context-dependent and may vary according to tumor subtype, stromal composition, disease stage, treatment exposure, and tissue site [3,7,11,31,32,33,34,54,55,56,76,77,78,79]. Importantly, the history of stromal and ECM-directed intervention shows that strong preclinical rationale does not necessarily translate into clinical efficacy, particularly when targets have pleiotropic or context-dependent functions, when patient-selection biomarkers are lacking, or when stromal modulation is approached as nonspecific depletion rather than microenvironmental normalization [80,81,82]. Therefore, therapeutic strategies aimed at the ECM should not be framed as broadly applicable anti-stromal interventions but as mechanism-guided approaches that require patient stratification, biomarker support, and careful validation.

Several ECM-associated therapeutic axes are biologically plausible. Inhibition of matrix crosslinking enzymes such as LOX and LOXL may reduce collagen stiffening, mechanotransduction, hypoxia-associated remodeling, and therapy resistance in selected tumor contexts [9,11,32,33,34,77]. Targeting integrin signaling, FAK/Src pathways, RhoA–ROCK-associated contractility, or YAP/TAZ-dependent mechanotransduction may interfere with the ability of tumor cells and stromal cells to sense and respond to a stiffened matrix [11,12,13,32,33,34,76,83]. Modulation of protease activity, including MMP-associated remodeling, may influence invasion, growth-factor release, vascular permeability, immune infiltration, and drug penetration [3,11,30,36,76,80]. Nevertheless, these pathways also participate in normal tissue remodeling, wound repair, vascular function, and immune regulation. This duality helps explain why ECM-targeted strategies may show strong mechanistic rationale but limited, context-dependent, or even unfavorable translational outcomes when applied without target specificity, biological stratification, and validation-oriented trial design [80,82].

Another therapeutic opportunity involves reshaping the tumor microenvironment to improve drug delivery and immune response. Dense collagen networks, stromal expansion, increased interstitial pressure, hypoxia, TGF-β-associated stromal programs, and matrix-driven immune exclusion can restrict therapeutic access and reduce responsiveness to systemic therapies [3,7,11,31,32,33,34,76,77,78,79,81,84]. In this setting, ECM modulation may be useful not as a standalone anticancer treatment but as a strategy to improve the effectiveness of chemotherapy, targeted therapy, immunotherapy, or drug-delivery systems [76,78,79,81,84]. For example, reducing excessive matrix stiffness or remodeling selected stromal barriers could theoretically improve perfusion, immune-cell infiltration, and therapeutic penetration. However, excessive stromal depletion may also remove tissue-restraining functions, alter immune balance, or promote more aggressive tumor behavior in specific settings [82]. Therefore, ECM normalization may be a more appropriate translational concept than nonspecific ECM ablation [81,82].

The relationship between ECM remodeling and immunotherapy is particularly relevant. Matrix stiffness, collagen organization, CAF-associated remodeling, protease activity, hypoxia, TGF-β signaling, and FAK-associated stromal programs can contribute to immune exclusion, altered antigen presentation, impaired T-cell infiltration, and immunosuppressive stromal niches [7,31,32,76,78,83,84]. These mechanisms suggest that ECM-targeted approaches could help convert selected immune-excluded tumors into more permissive microenvironments. However, this possibility remains highly dependent on tumor type, immune contexture, stromal phenotype, and treatment combination. For ECM-centered immunomodulatory strategies to become clinically meaningful, candidate matrix targets should be linked to measurable biomarkers, spatial immune organization, response endpoints, and validation in appropriately designed cohorts.

Spontaneous canine mammary tumors may contribute to this translational field by providing naturally occurring disease contexts in which ECM remodeling, stromal heterogeneity, immune–matrix interactions, and treatment-response patterns can be investigated under less reductionist conditions than many experimental models [17,18,19,20,21,22,23,37,40,41,42,43,44,45,46,47,50,53]. Their greatest value is not to serve as direct substitutes for human trials, but to help prioritize which ECM-associated mechanisms deserve further investigation across species. For example, canine tumors with collagen-rich stroma, CAF-associated remodeling, protease activity, immune exclusion, or altered ECM protein patterns may be useful for testing whether selected matrix phenotypes are associated with therapeutic resistance, metastatic behavior, or treatment-response variability. Such studies, however, require standardized pathology, defined treatment regimens, longitudinal follow-up, and careful control of histological subtype and clinical stage.

Overall, ECM-targeted therapy should be viewed as an emerging and validation-dependent translational direction rather than a mature clinical framework [76,77,78,79,80,81,82,83,84]. The most realistic near-term application may be the use of ECM-centered biomarkers to identify matrix-defined tumor states, stratify patients or animal cohorts for mechanistic studies, and prioritize rational combinations with existing therapies. In this context, multi-omics, spatial profiling, digital pathology, and AI-assisted modeling can support therapeutic translation only when they are connected to reproducible biomarkers, biologically plausible mechanisms, and clinically interpretable endpoints. This integrated, cautious approach is essential for advancing ECM-centered oncology from descriptive matrix biology to actionable, rigorously validated therapeutic strategies.

## 6. Conclusions

ECM remodeling is a central mechanobiological component of breast cancer aggressiveness. Rather than functioning only as a passive scaffold or structural consequence of tumor growth, the tumor-associated ECM contributes to stromal permissiveness, invasion, mechanotransduction, immune modulation, metabolic adaptation, therapeutic resistance, and metastatic progression. These effects arise from coordinated interactions among matrix composition, collagen architecture, tissue stiffness, remodeling enzymes, stromal cells, immune compartments, vascular organization, and tumor-cell signaling pathways. Therefore, ECM-associated features should be interpreted as integrated matrix-defined phenotypes rather than isolated molecular or structural markers.

Spontaneous canine mammary tumors provide a complementary comparative context for investigating ECM-informed mechanisms under naturally occurring disease conditions. Their relevance lies in their ability to preserve aspects of tumor–stroma–ECM coevolution within immunocompetent hosts, rather than in direct equivalence to human breast cancer. Cross-species comparisons are most informative when they focus on partially conserved stromal programs, such as collagen-rich remodeling, CAF-associated matrix deposition, protease activity, immune–matrix crosstalk, invasive-front organization, and alterations in ECM protein patterns. However, species-specific differences in tumor subtype distribution, hormone receptor status, reproductive history, clinical management, histopathological classification, tissue processing, and treatment exposure require cautious interpretation.

ECM-focused proteomics, matrisome-informed profiling, lipidomics, metabolomics, spatial omics, digital pathology, and AI-assisted modeling offer important opportunities for biomarker discovery, risk stratification, biological prioritization, and response prediction. However, these approaches do not overcome biological and methodological limitations on their own. Candidate ECM signatures should be evaluated according to analytical validity, biological plausibility, clinical validity, clinical utility, and incremental value beyond established prognostic factors. Their translational value depends on standardized pathology, reproducible assays, harmonized metadata, interpretable models, external validation, and clinically meaningful endpoints.

Therapeutically, the ECM represents an important but complex translational target. Matrix crosslinking, mechanotransduction, protease activity, stromal activation, immune exclusion, metabolic adaptation, and impaired drug penetration provide biologically plausible intervention axes. Nevertheless, ECM-targeted strategies require careful patient or cohort stratification because matrix components can have context-dependent and sometimes tumor-restraining functions. The most realistic near-term direction may not be nonspecific stromal depletion, but mechanism-guided ECM normalization, rational therapeutic combinations, and biomarker-supported identification of matrix-defined tumor states. Overall, ECM-informed comparative oncology should advance as a disciplined framework grounded in rigorous validation, in which canine models support hypothesis generation and biological prioritization while human breast cancer datasets remain essential for clinical validation and translation.

## Figures and Tables

**Table 1:** Major ECM molecular and functional categories associated with breast cancer aggressiveness.

ECM Molecular/Functional Category	Representative Molecules	Main ECM/TME Function	Aggressiveness-Related Output	Major Signaling or Functional Axes
Collagenous network	Collagens I, III, and IV	Regulate matrix stiffness, basement membrane integrity, tissue compartmentalization, and invasion routes	Increased stiffness, directional invasion, basement membrane disruption, EMT, and poor clinical outcome	Integrins/FAK/Src; RhoA–ROCK; YAP/TAZ; PI3K–AKT; MAPK [2,8,9,10,11,32,33]
Adhesive glycoproteins	Fibronectin	Promote integrin-dependent adhesion, focal adhesion assembly, and cooperation with collagen-rich matrices	Enhanced migration, invasion, EMT-associated phenotypes, and metastatic progression	Integrin α5β1/FAK/Src; ERK; TGF-β; Wnt/β-catenin [2,4,11,33]
Basement membrane glycoproteins	Laminin isoforms	Regulate epithelial polarity, basement membrane organization, adhesion, and angiogenic signaling	Loss of epithelial organization, altered adhesion, invasion, and vascular remodeling	Integrins α6β4/α3β1; PI3K–AKT; MAPK; context-dependent Hippo/YAP/TAZ signaling [2,4,11]
Elastic fiber-associated components	Elastin and elastin-derived fragments	Contribute to tissue compliance, vascular remodeling, and protease-associated matrix remodeling	Increased migration, angiogenesis, and protease activity in selected tumor contexts	EGFR/ERK; PI3K–AKT; VEGF induction; MMP-associated remodeling [2,4,11]
Proteoglycans with predominantly tumor-restraining functions	Decorin and lumican	Regulate collagen fibrillogenesis, growth-factor availability, and stromal organization	Context-dependent restriction of proliferation, angiogenesis, and invasion	TGF-β/SMAD modulation; EGFR/MET regulation; collagen organization; antiangiogenic signaling [2,11,35]
Proteoglycans frequently associated with tumor-promoting microenvironments	Biglycan, versican, perlecan, glypicans, and CSPG4	Regulate hydration, growth-factor reservoirs, inflammation, fibroblast activation, and receptor engagement	CAF activation, angiogenesis, migration, invasion, immune modulation, and stromal expansion	TLR2/4–NF-κB; VEGF/FGF signaling; PI3K–AKT; MAPK; Wnt/β-catenin; TGF-β [2,11,35]
Matricellular signaling regulators	Tenascin-C, periostin, osteopontin, SPARC, and thrombospondins	Act as context-dependent modulators of cell–matrix adhesion, collagen remodeling, CAF activation, angiogenesis, and immune modulation	Formation of invasive niches, metastatic colonization, altered stromal mechanics, and therapy-relevant microenvironmental remodeling	Integrins/FAK/Src; TGF-β; Wnt/β-catenin; receptor tyrosine kinases; angiogenic and immune-regulatory pathways [14,15,24]
ECM-degrading enzymes	MMPs, heparanase, and related proteases	Degrade basement membrane and interstitial ECM, generate invasion tracks, release growth factors, and produce bioactive ECM fragments	Local invasion, angiogenesis, vascular permeability, immune infiltration, metastatic dissemination, and altered drug penetration	MMP-dependent proteolysis; VEGF/FGF/TGF-β release; inflammatory signaling; invasion-track formation [3,11,30,36]
ECM-crosslinking enzymes	LOX and LOXL family enzymes	Promote collagen crosslinking, matrix stiffening, and mechanical reinforcement of tumor stroma	Increased mechanotransduction, invasion, hypoxia-associated remodeling, and therapy resistance	Integrin clustering; FAK/Src; RhoA–ROCK; YAP/TAZ; stiffness-driven feedback loops [9,11,32,33,34]

Abbreviations: CAF: cancer-associated fibroblast; CSPG4: chondroitin sulfate proteoglycan 4; ECM: extracellular matrix; EGFR: epidermal growth factor receptor; EMT: epithelial–mesenchymal transition; ERK: extracellular signal-regulated kinase; FAK: focal adhesion kinase; FGF: fibroblast growth factor; LOX: lysyl oxidase; Src: Src-family tyrosine kinases; LOXL: lysyl oxidase-like; MAPK: mitogen-activated protein kinase; MET: mesenchymal–epithelial transition factor receptor; MMP: matrix metalloproteinase; NF-κB: nuclear factor kappa B; PI3K–AKT: phosphoinositide 3-kinase–protein kinase B pathway; RhoA–ROCK: Ras homolog family member A–Rho-associated coiled-coil-containing protein kinase pathway; SMAD: mothers against decapentaplegic homolog; SPARC: secreted protein acidic and rich in cysteine; TGF-β: transforming growth factor beta; TLR: Toll-like receptor; TME: tumor microenvironment; VEGF: vascular endothelial growth factor; Wnt: wingless-related integration site signaling pathway; YAP/TAZ: Yes-associated protein/transcriptional coactivator with PDZ-binding motif.

## Data Availability

No new data were created or analyzed in this study. Data sharing is not applicable to this article.
